# Prehabilitation of Patients With Oesophageal Malignancy Undergoing Peri‐Operative Treatment (Pre‐EMPT): Outcomes From a Prospective Controlled Trial

**DOI:** 10.1002/jso.28079

**Published:** 2025-01-29

**Authors:** R. Bott, J. Zylstra, W. Knight, G. P. Whyte, A. M. Lane, C. Moss, M. Browning, J. Lagergren, M. Van Hemelrijck, A. R. Davies, the Pre‐EMPT study group including the following co‐authors, T. Dixon, G. Hallward, C. Taylor, N. Maisey, S. Ngan, A. Lumsden, K. Owczarczyk, A. Qureshi, N. Griffin, A. Jacques, V. Goh, M. Green, H. Deere, F. Chang, U. Mahadeva, B. Gill‐Barman, M. Ong, S. George, J. Dunn, S. Zeki, J. Waters, M. Cominos, T. Sevitt, M. Hill, A. Santa Ollala, K. Beckmann, L. Gervais‐Andre, J. Pate, J. Gossage, M. Kelly, C. Baker, J. Taylor, O. Rusu, O. Evans, G. Tham

**Affiliations:** ^1^ Department of Upper GI and General Surgery Guy's and St Thomas' NHS Foundation Trust London United Kingdom; ^2^ Sport and Exercise Sciences, Faculty of Science Liverpool John Moore's University Liverpool United Kingdom; ^3^ Centre for Health and Human Performance London United Kingdom; ^4^ University of Wolverhampton Research Centre for Sport, Exercise and Performance (RCSEP) Wolverhampton United Kingdom; ^5^ School of Cancer and Pharmaceutical Sciences King's College London London United Kingdom; ^6^ Department of Anaesthesia and Intensive Care Maidstone and Tunbridge Wells NHS Trust Maidstone United Kingdom; ^7^ Department of Molecular Medicine and Surgery Karolinska Institutet Stockholm Sweden; ^8^ Department of Anaesthesia Guy's and St Thomas' NHS Foundation Trust London United Kingdom; ^9^ Department of Oncology Guy's and St Thomas' NHS Foundation Trust London United Kingdom; ^10^ Department of Radiology Guy's and St Thomas’ NHS Foundation Trust London United Kingdom; ^11^ Cancer Imaging, School of Biomedical Engineering and Imaging Sciences King's College London London United Kingdom; ^12^ Department of Pathology Guy's and St Thomas’ NHS Foundation Trust London United Kingdom; ^13^ Department of Gastroenterology Guy's and St Thomas’ NHS Foundation Trust London United Kingdom; ^14^ Department of Oncology Maidstone and Tunbridge Wells NHS Trust Maidstone UK; ^15^ Comprehensive Cancer Centre King's College London London United Kingdom

**Keywords:** cardiopulmonary exercise testing, health‐related quality of life, neoadjuvant chemotherapy, oesophageal adenocarcinoma, prehabilitation

## Abstract

**Background:**

The Pre‐EMPT study aimed to determine if structured exercise could reduce length of stay, post‐operative complications and improve fitness and health‐related quality of life (HQRL) in patients undergoing neoadjuvant chemotherapy (NAC) and oesophagectomy.

**Methods:**

A prospective non‐randomised trial compared a standard care pathway (control) to a structured prehabilitation exercise programme (intervention) commenced before NAC and surgery for oesophageal adenocarcinoma. Length of hospital stay and post‐operative complications were recorded. Cardiopulmonary exercise testing (CPEX), body composition analyses, lymphocyte levels and HRQL questionnaires were performed at multiple time points.

**Results:**

Median length of stay was similar in both groups. There were 6 versus 11 complications observed (intervention vs control *p* = 0.086). Cardio‐pulmonary fitness (VO2peak) declined after NAC, but less in the intervention group (intervention −13.54% vs control −21.40%, *p* = 0.02). Body composition improved in the intervention group (FMi/FFMi −5.5% intervention, 10.7% control *p* = 0.043). Performance, cognitive, sleep and emotional function scores improved following NAC in the intervention group. Lymphocyte subsets increased in the intervention group compared to the control group after chemotherapy (*p* = 0.034). Chemotherapy response was improved in the intervention group (*p* = 0.022).

**Conclusion:**

A structured exercise programme may mitigate cardiopulmonary deconditioning, reduce sarcopenia and offset lymphopenia, during chemotherapy, in patients undergoing NAC and oesophagectomy.

## 1 Introduction

Neoadjuvant chemotherapy (NAC) or chemoradiotherapy followed by oesophagectomy currently offers the best chance of cure for suitable patients with invasive adenocarcinoma of the oesophagus [1, 2, 3]. It is, however, a demanding treatment regimen, involving a high risk of peri‐operative morbidity [4]. Pre‐operative oncological treatments have a substantial negative impact on a patient's cardiovascular fitness, muscle mass and health‐related quality of life (HRQL) even before the considerable insult of an oesophagectomy [4, 5, 6, 7, 8, 9, 10].

Pre‐operative exercise as part of a multi‐modal prehabilitation programme has shown promising results in reducing post‐operative complications in abdominal surgery [11] and following major cancer resections [12]. A recent study in oesophagectomy patients showed a reduction in post‐operative pneumonia rates and length of stay in patients undergoing prehabilitation [13]. The extent to which prehabilitation mitigates neoadjuvant treatment‐associated physical and psychological deconditioning prior to surgery is still being explored.

The purpose of this study was to evaluate the impact of a structured prehabilitation exercise programme started prior to and continued during NAC, up to oesophagectomy, in patients with operable oesophago‐gastric cancer compared with those on a standard treatment pathway. This study reports the primary and secondary outcome measures of the trial.

## 2 Materials and Methods

### 2.1 Trial Design

Following patient consultation in collaboration with the Oesophageal Patients Association, ethical approval was granted (REC no. 16/SC/0438; ClinicalTrials.gov: NCT03626610). The trial was entitled ‘Prehabilitation of patients with oEsophageal Malignancy undergoing Peri‐operative Treatment’ (Pre‐EMPT). It was a non‐randomised, interventional study that assessed the effects of a structured exercise programme, or ‘prehabilitation’, in patients undergoing NAC for adenocarcinoma of the lower oesophagus or gastro‐oesophageal junction'. The study compared patients undergoing a conventional treatment pathway, including specialist dietetic input (control group), with the addition of a structured exercise programme (intervention group).

Guy's and St Thomas NHS Foundation Trust receives upper gastrointestinal cancer referrals for surgery from two similar‐sized cancer networks. Patients were invited to participate in the trial intervention or control arms depending on their network of origin. Randomisation into treatment arms was considered at the outset, however, the exercise intervention was delivered by a collaborating institution in London. As a result, the time and financial constraints for patients regularly travelling from the more distant geographical region to receive the intervention were considered too excessive. Clinical commissioning constraints also mandated a minimisation of travel to the centre from patients within this region. Audits conducted prior to the study demonstrated similar demographics, staging, treatment pathways and outcomes for patients from both regions.

All patients were discussed in a centralised specialist upper gastro‐intestinal multi‐disciplinary meeting and underwent a standard pathway of staging investigations including oesophago‐gastro‐duodenoscopy and tissue biopsy, computed tomography imaging, positron emission tomography imaging and endoscopic ultrasound. Staging laparoscopy was used selectively. All patients who were deemed surgical candidates were assessed and operated on at the surgical centre.

**Figure 1:**
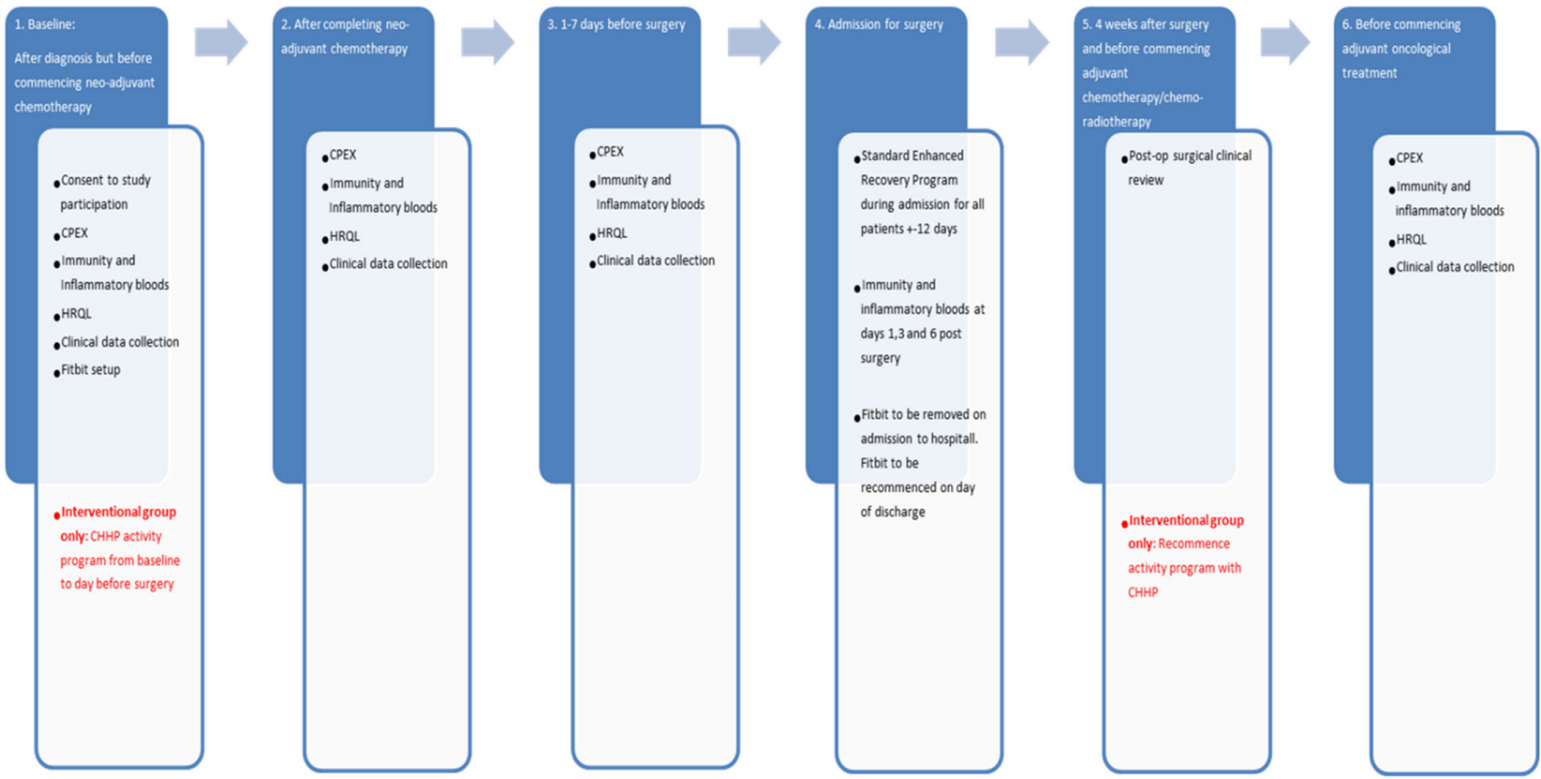
Pre‐EMPT Trial Flowchart.

Patients underwent informed and written consent prior to trial participation and undertook baseline study procedures as per study protocol (Figure 1). Patients in both groups received nutritional, physical activity and smoking cessation advice from specialist nurses, physiotherapists, and dieticians, as is standard practice for all patients on the oesophageal surgical‐oncological pathway. Patients in the intervention group attended additional exercise sessions with a specialist Exercise Physiologist, at the Centre for Health and Human Performance in London. They were also provided with written and diagrammatic instructions on how to continue the exercise programme at home. The prehabilitation exercise programme undertaken was based on the World Health Organisation and Macmillan ‘Recommended levels of physical activity for adults aged 18– 64 years, “also relevant to healthy adults aged 65 and above,” unless contraindicated’ guidelines, incorporating combined aerobic and strength training (WHO and MacMillan).

Initial trial measures were taken at baseline and within 1 week of completion of NAC. Patients were then reviewed in the multidisciplinary meeting for suitability for surgery. Further sets of trial measures were carried out during the week prior to surgery, post‐operatively and before commencement of adjuvant treatment (Figure 1).

### 2.2 Oncological and Surgical Treatment

With the publication of the FLOT trial [3], oncological practices changed in both cancer networks during the study. At first, patients received epirubicin, cisplatin and 5 flurouracil (ECF) or epirubicin, oxaliplatin and capecitabine (ECX) with three cycles of chemotherapy before and after surgery. In the latter half of the study, patients received four cycles of pre and post‐operative FLOT. The study protocol accommodated this change in practice. Oesophagectomy included transhiatal or transthoracic resections at the discretion of the individual surgeon taking into account patient and tumour characteristics. The clinical team were blinded to the group allocation of each patient.

### 2.3 Outcome Measures

Primary outcome measures were length of stay (LOS) and surgical complications (Clavien‐Dindo classification). Initial power calculations intended to recruit 68 patients (34 in each group), which permitting a 10% dropout rate, would have allowed a comparison of 62 patients (31 in each group) aiming to show a LOS reduction from 12 to 10 days and a reduction in complications from 50% to 25%.

### 2.4 Cardiopulmonary Exercise Testing (CPEX)

Peak oxygen uptake (VO_2peak_, ml. kg^−1^. min^−1^) and anaerobic threshold (AT, ml. kg^−1^. min^−1^) were recorded at baseline, following NAC and before surgery. The Ergoline Ergoselect 200 cycle ergometer was used in the control group and an Ergoline 900 model cycle ergometer was used in the intervention group, both incorporating a ramp protocol. The ramp speed in Watts. min^−1^ was selected based on predicted VO_2_ and adjusted to the individual.

### 2.5 Body Composition

Computed tomography scans of the thorax and abdomen were performed routinely on all patients at baseline and following NAC. In each participant, axial images equivalent to a 10 mm z‐axis stack were sampled at the level of the third lumbar vertebra. The standard Digital Imaging and Communications in Medicine (DICOM) images were assessed using in‐house software (King's College London) with fat and muscle tissue segmentation performed by a radiologist. Following Hounsfield unit thresholding and automated segmentation of the subcutaneous and visceral fat and skeletal muscle at the L3 level, parameters including fat‐to‐muscle ratio (FMR), fat‐free mass index (FFMi) and fat mass index (FMi) were assessed [10, 14, 15].

### 2.6 Health‐Related Quality of Life

The trial employed two validated questionnaires: A cancer‐specific questionnaire, EORTC QLQ‐ C30 [16] and the shortened Warwick‐Edinburgh Mental Well‐being scale (SWEMWBS) [17] to evaluate patient‐reported outcomes (PROMS). The EORTC QLQ C‐30 questions and their functional grouping are shown in Table S1. Patients were asked to complete these self‐reported questionnaires at baseline, after NAC, before and after surgery and at 6 and 12 months after surgery. EORTC QLQ‐C30 (Quality of Life of Cancer Patients) and the SWEMWBS questionnaires were selected following registration with the relevant organisations. Average scores were compared at different time points and T‐tests were used to test for the difference in means between the intervention and control groups for changes from baseline to the various time points.

### 2.7 Bloods

T‐lymphocyte subsets were analysed using Laser Flow Cytometry of a monoclonal antibody/blood sample reaction on a Beckman Coulter AQUIOS flow cytometer (BECKMAN COULTER Life Sciences, 5350 Lakeview Parkway S Drive, Indianapolis, IN 46268, USA). Red cells were lysed through the addition of a lysing solution. A stream of the remaining single cells of antibody‐antigen reaction was passed through a laser beam interrogation point. The emitted light passes through wavelength filters separating out the subset components. The results were produced as a series of histogram plots and analysed according to grouping. Manual ‘gating’ was carried out by an Immunologist for quality control.

## 3 Results

### 3.1 Patient Demographics

This study analysed 21 patients in the intervention group and 20 patients in the control group who completed NAC. Although 62 patients were recruited, 21 patients dropped out by failing to complete the required pathway and therefore did not satisfy the inclusion criteria. Trial recruitment was hindered by the outbreak of the COVID‐19 pandemic. There were comparable baseline demographics of age, sex and tumour characteristics between the two groups (Table 1). All patients completed NAC with 6 patients requiring a dose reduction. One patient in each group suffered disease progression during NAC and did not proceed to resection.

**Table 1:** Characteristics of study patients with oesophageal adenocarcinoma.

Variables		Intervention	(%)	Control	(%)	*p* value
		group		group		
Number of participants at baseline		21		20		
Age (years; median)		63		65		0.834
Sex (M:F)	Male	17	81.0	18	90	0.413
	Female	4	19.0	2	10	
Median age‐adjusted Charlson Comorbidity score		2		2		
Mean age‐adjusted Charlson Comorbidity score		1.9		2.1		0.645
BMI (kg.m^2^; median		26.2		28		0.401
Mean		25.3		26		
Clinical tumour stage	T3‐4	18	85.7	19	95	0.317
	N +	20	95.2	18	90	0.520
Neo‐adjuvant chemotherapy regimen	FLOT	11	52.4	8	40	0.427
	Other	10	47.6	12	60	
Neoadjuvant chemotherapy completed	Yes	21	100.0	19	95	
	Dose reduced	6	28.6	2	10	0.133
Post op stage	CPR	2	9.5	1	5	
	T1‐2	12	57.1	5	25	0.08
	T3‐4	7	33.3	14	70	0.012
	N0	11	52.4	6	30	0.146
	N1	7	33.3	5	25	0.558
	N2,3	3	14.3	9	45	0.031
Circumferential resection margin positivity		5	23.8	6	30	0.655
Mandard 1,2		7	33.3	1	5	0.022
Median Length of stay		10.5		11		0.263
Postoperative complications		8	38.1	11	55	0.277
Clavien Dindo 1,2		5	23.8	5	25	0.929
Clavien Dindo 3a		0	0.0	5	25	
Clavien Dindo 3b		1	4.8	1	5	
Clavien Dindo 4		1	4.8	0	0	
Clavien Dindo 5		1	4.8	0	0	
			0.0		0	
Anastomotic leak		1	4.8	3	15	0.269
Recurrence		5	23.8	4	20	0.907

### 3.2 Length of Stay and Complications

Hospital length of stay was lower in the intervention group although this did not reach statistical significance (intervention 10.5 days vs control 11 days, *p* = 0.263). Overall complication rates favoured the intervention group, again not statistically significant (intervention 8/21 (38.0%) vs control 11/20 (55%) (*p* = 0.277)). The rate of minor to moderate complications not requiring surgical intervention favoured the intervention group (Clavien‐Dindo 1‐3 intervention 6/21 (28.6%) vs control 11/20 (55%), *p* = 0.086). In the intervention group, there was one post‐operative death in a patient who suffered an aspiration‐related respiratory arrest on Day 10 post‐operatively having been scheduled for discharge the following day. Also in the intervention group was one patient who returned to theatre for an irreducible (pre‐existing) umbilical hernia on Day 5 following surgery, requiring open repair. This was unrelated to the laparoscopic port site.

### 3.3 Cardio‐Pulmonary Fitness

There was a marked deterioration in AT and VO_2peak_ in both groups after NAC. The control group experienced a mean decline of 20.2% in AT and 21.4% in VO_2peak_. The intervention group experienced a less marked deterioration of 13.4% AT (*p* = 0.101) and 13.5% VO_2peak_ (*p* = 0.02). VO_2peak_ levels recovered prior to surgery (−8.51% from baseline in the intervention group and ‐11.3% in the control group (*p* = 0.413) (Table 2; Figure S1).

**Table 2:** CPEX outcomes by treatment group at baseline post neoadjuvant chemotherapy (NAC) for oesophageal adenocarcinoma.

		Intervention group			Control group			
		Mean (ml. kg^‐1^. min^‐1^)	Mean difference	% Difference	Mean (ml. kg^1^. min^‐1^)	Mean difference	% Difference	*p*‐value
Anaerobic threshold	Baseline	15.92 (12.85–18.32)			14.44 (12.25–15.20)			
	Post‐NAC	13.94 (12.22–15.07)	−2.49	−13.38%	12.21 (10.15–13.53)	−3.34	−20.20%	*p* = 0.101
	Pre‐surgery	14.54 (13.38–16.01)	−2.45	−12.98%	12.98 (11.40–14.25)	−1.79	−10.19%	*p* = 0.412
VO2peak	Baseline	24.85 (21.38–28.05)			22.96 (19.48–26.05)			
	Post‐NAC	22.23 (18.45–26.75)	−3.62	−13.54%	18.99 (17.52–21.47)	−5.47	−21.40%	**p** = **0.02**
	Pre‐surgery	24.38 (20.98–28.09)	−2.40	−8.51%	21.05 (18.53–23.85)	−2.87	−11.34%	*p* = 0.413

### 3.4 Body Composition

Median fat‐to‐muscle ratio (FMR) improved in the intervention group (−10.9%) after prehabilitation, with increases in skeletal muscle and decreases in visceral and subcutaneous fat areas compared to baseline scans. Fat‐free mass index (FFMi) and fat mass index (FMi), normalised for patient height, also improved in the intervention group (median FFMi Intervention +4.7% *vs*. −3.2% Control, *p* = 0.0596), as well as FMI/FFMI (−5.8% intervention vs 12.2% control, *p* = 0.04).

Median FMR in the control group increased by 12.4% with overall weight gain associated with increased visceral and peripheral fat, and reduced muscle mass. There was a corresponding increase in overall fat mass in the control group, especially visceral fat (median FMI increase of −0.8% intervention *vs*. 3.8% control, *p* = 0.192), but not statistically significant (Table 3).

**Table 3:** Body composition before and after neoadjuvant chemotherapy (NAC) for oesophageal adenocarcinoma by treatment groups. FM (fat mass), FMI (fat mass index), FFM (fat‐free mass), FFMI (fat‐free mass index), FMR (fat mass ratio).

(*n* = 22 and 20)	Intervention	Control	
**Parameters**	**Mean (interquartile range)**	**Mean (interquartile range)**	***p*** **‐value**
**FFM index (kg/m** ^**2**^ **)**			
Baseline	16.12 (13.04–10.82)	15.43 (13.32–18.17)	
Post‐treatment	17.33 (13.30–19.81)	14.84 (12.91–17.46)	
Change (%)	7.51% (‐5.76–28.90)	−3.87% (‐15.79–8.92)	0.0596
**FM index (kg/m** ^**2**^ **)**			
Baseline	9.26 (6.80–10.82)	8.35 (7.87–10.31)	
Post‐treatment	9.11	8.44	
	8.86 (6.86–10.91)	(7.74‐10.36)	
Change (%)	0.00% (‐9.54–6.00)	2.71% (4.18–8.84)	0.192
**FMI/FFMI**			
Baseline	0.65 (0.42–0.76)	0.55 0.51 (0.38–0.61)	
Post‐treatment	0.57 (0.39–0.73)	0.58 0.58 (0.41–0.68)	
Change (%)	‐5.51% (‐30.80–9.92)	10.74% ‐12.15% (‐8.40–19.41)	0.043
**FMR**			
Baseline	1.70 (0.73–2.26)	1.20 0.99 (0.65–1.35)	
Post‐treatment	1.43 (0.65–1.95)	1.26 1.08 (0.70–1.48)	
Change (%)	0.17% (‐35.95‐33.68)	20.24% (‐10.23 ‐26.23)	0.134
**Visceral fat (cm** ^**2**^ **)**			
Baseline	189.27 (54.53–257.49)	193.15 (111.60–260.58)	
Post‐treatment	184.23 (75.81–273.71)	200.52 (137.76–247.28)	
Change (%)	10.13% (‐21.32‐19.42)	22.70% (‐3.48‐26.17)	0.1344
**Subcutaneous fat (cm** ^**2**^ **)**			
Baseline	191.09	164.16	
	167.18 (114.15–277.31)	136.00 (111.41–172.24)	
Post‐treatment	187.21	164.01	
	163.91 (112.09–248.13)	144.94 (112.13–174.08)	
Change (%)	6.47%	11.77%	0.455
	‐3.70% (‐11.86–15.09)	2.48% (‐5.92–9.44)	
**Subcutaneous muscle (cm** ^**2**^ **)**			
Baseline	136.75 (106.59–167.83)	141.63 155.35 (109.61–163.51)	
Post‐treatment	149.59 (112.83–176.76)	135.08 137.62 (114.78–157.37)	
Change (%)	19.41% (‐6.74–33.36)	‐1.07% ‐3.57% (‐17.82–10.52)	0.063
**VA/SA ratio**			
Baseline	1.50 (0.70–1.83)	1.13 0.84 (0.68–1.15)	
Post‐treatment	1.45 (0.70–1.80)	0.94 0.80 (0.65–1.02)	
Change (%)	2.10% (‐15.61–19.54)	6.77% ‐4.78% (‐13.92–1.44%)	0.063
**BMI (kg/m** ^**2**^ **)**			
Baseline	25.66 (22.20–29.00)	26.33 27.43 (22.11–29.10)	
Post‐treatment	25.68 (22.91–29.21)	26.95 27.11 (23.25–30.22)	
Change (%)	0.16% (‐3.11–4.65)	2.63% 2.60% (0.00–4.37)	0.052

### 3.5 Health‐Related Quality of Life

**Figure 2:**
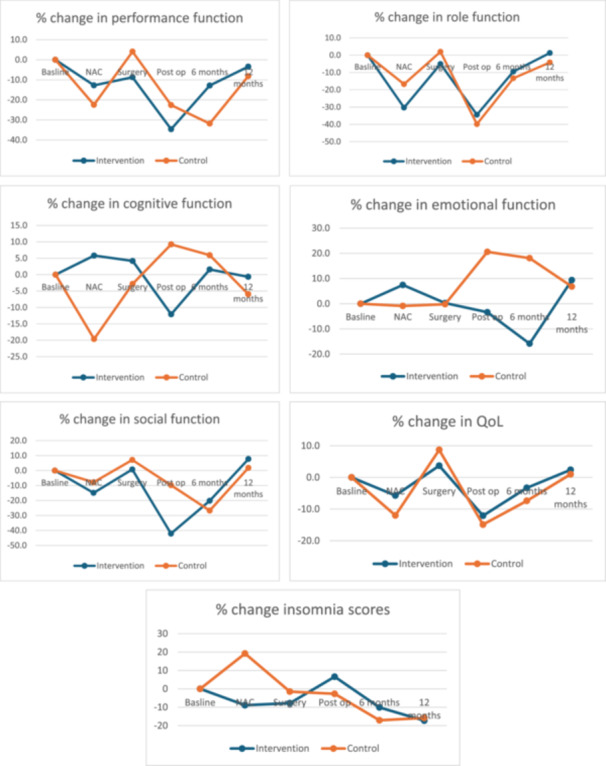
EORTC cognitive, performance and emotional functioning and quality of life at baseline, post NAC and pre‐surgery.

HRQL results are summarised in Table 4 and Figure 2. During NAC, physical functioning declined in both groups but more so in the control group (−12.8% vs ‐22.5% *p* = 0.85). There was an improvement in cognitive and emotional function in the intervention group (5.8% vs −19.6% *p* = 0.62, 7.4% vs −0.9% *p* = 0.54) following chemotherapy. Quality of life scores fell in both groups after neo‐adjuvant chemotherapy but less so in the intervention group (−5.7% vs −12% *p* = 0.77). Quality of life scores were also marginally higher in the post‐operative period and at 6 months and 12 months post‐operative in the intervention group (6‐month; intervention −3.3% and control −7.4%, 12‐month intervention 2.4% and control 1%). Patients in the intervention group reported worse social and role function during treatment although these scores improved at 12 months post‐operatively when compared to the control group. Emotional function fell significantly in the intervention group post‐operatively and at 6 months compared to the control group (post‐op intervention −3.4% control 20.6% *p* = 0.07; 6 months intervention −15.8 vs control 18.1 *p* = 0.01).

**Table 4:** Percentage change in HRQL scores by treatment group at specified study time points.

EORTC question group		% change from baseline									
		NAC	p value	Pre‐op	p value	Post‐op	p value	6 months	p value	12 months	*p* value
Performance functioning	Intervention	−12.8	0.85	−8.7	0.23	−34.6	0.98	−12.8	0.69	−3.4	0.59
	Control	−22.5		4.1		−22.6		−31.8		−8.2	
Role functioning	Intervention	−30.3	0.12	−5.1	0.50	−34.4	0.98	−9.5	0.89	1.3	0.69
	Control	−16.8		2.0		−39.8		−13.3		−4.2	
Cognitive functioning	Intervention	5.8	0.62	4.2	0.08	−12.1	0.81	1.6	0.59	−0.7	0.59
	Control	−19.6		−2.8		9.2		5.9		−6.0	
Emotional functioning	Intervention	7.4	0.54	0.3	0.76	−3.4	0.07	−15.8	0.01	9.4	0.68
	Control	−0.9		−0.2		20.6		18.1		6.8	
Social functioning	Intervention	−14.9	0.37	0.8	0.82	−42.1	0.35	−20.2	0.74	7.7	0.78
	Control	−7.8		7.1		−9.8		−26.7		1.8	
Quality of life	Intervention	−5.7	0.77	3.7	0.97	−12.1	0.87	−3.3	0.98	2.4	0.53
	Control	−12.0		8.7		−14.9		−7.4		1.0	
Difficulty sleeping	Intervention	−8.9	0.13	−7.8	0.10	6.6	0.63	−10.1	0.74	−17.3	0.60
	Control	19.2		−1.5		−2.7		−17.1		−15.8	

### 3.6 Lymphocytes

CD3, CD4 and CD8 lymphocyte concentration and mean changes of concentrations at the seven time points are shown in Table 5. Concentrations of CD3, CD4, CD8 lymphocytes at baseline were similar in the intervention and control groups (CD3 1154.35 and 1151.45, CD4 711.6 and 693.2, CD8 427.35 and 445.15). All lymphocytes increased significantly in the intervention group after chemotherapy (CD3 84.92% vs 10.92% *p* = 0.015, CD4 107.75% vs 13.27% *p* = 0.0147, CD8 69% vs 12% *p* = 0.033). Mean % changes in lymphocyte concentration over the seven‐time points are shown in Figure S2.

**Table 5:** Lymphocyte levels by treatment groups at specified study timepoints.

	CD3		CD4		CD8		CD4/8	
	Intervention	Control	Intervention	Control	Intervention	Control	Intervention	Control
Baseline	1154.35	1151.45	711.6	693.2	427.35	445.15	2.03	1.94
Post NAC	1484.44	1148.00	959.22	728.53	498.00	399.27	2.28	2.09
% change from baseline	84.92%	10.92%	107.75%	13.72%	69.43%	12.22%	28.13%	4.04%
p‐value	0.015		0.016		0.034		0.4646	
Admission	1297.38	1283.05	826.67	799.00	450.86	452.15	2.23	2.02
% change from baseline	58.01%	15.58%	80.24%	19.77%	45.09%	10.91%	27.95%	6.64%
p‐value	0.784		0.992		0.711		0.283	
Day 1	651.86	740.84	412.29	435.00	234.19	296.84	2.00	1.81
% change from baseline	—	−30.10%	−9.78%	−30.61%	−18.22%	−27.91%	20.84%	−0.70%
p‐value	0.565		0.810		0.881		0.6673	
Day 3	674.74	737.06	431.11	444.11	238.32	274.22	2.14	1.97
% change from baseline	−27.19%	−28.04%	−16.33%	−26.24%	−33.84%	−31.94%	25.19%	−5.95%
p‐value	0.689		0.459		0.960		0.17068	
Day 6	849.59	794.45	556.06	515.80	284.47	266.25	2.29	2.18
% change from baseline	−14.03%	−26.78%	−15.47%	−23.09%	−21.04%	−33.15%	18.10%	16.48%
p‐value	0.741		0.826		0.596		0.92828	
Week 6	876.18	1152.87	519.59	643.27	301.82	461.53	2.19	1.69
% change from baseline	−45.42%	11.82%	−2.96%	4.97%	−12.08%	22.72%	−8.27%	−32.06%
p‐value	0.142		0.327		0.013		0.13104	

### 3.7 Chemotherapy Response

A significantly improved chemotherapy response was observed in the intervention group when using the Mandard Tumour Regression Grade and dividing into two subgroups groups: Mandard 1 and 2 versus Mandard 3 to 5 (intervention 33% Mandard 1&2 vs control 5% Mandard 1&2, *p* = 0.02).

## 4 Discussion

This trial did not show a statistically significant difference in hospital length of stay or overall post‐operative complication rates as a result of prehabilitation, although both were reduced in the intervention group. The results indicate that a structured exercise programme mitigates the decline in physical fitness associated with neoadjuvant chemotherapy. Patients in the intervention group also experienced a reversal of sarcopenia and sarcopenic obesity and a reduction in the detrimental impact of chemotherapy on patient HRQL was observed. Patients undertaking exercise showed significantly higher levels of lymphocytes during NAC, suggesting a boost in tumour‐related immunity during this time, and an improvement in chemotherapy response rates. Larger studies will be needed to determine if these benefits translate into a reduction in morbidity and mortality.

This prospective trial has shown some novel and statistically significant findings; however, some methodological issues warrant discussion. The statistical power was limited because of the reduced sample size created by a higher than expected dropout rate and the impact of the COVID‐19 pandemic. Given the patient numbers, the findings of this study still need to be confirmed in a larger study. The trial, whilst prospective, was not randomised for the reasons outlined above. As a result, it is difficult to completely eliminate some bias from the analysis. However, patients in the two geographical regions had similar demographics and staging at diagnosis and received equivalent treatment with the exception of the exercise intervention. One potential criticism of this study is that patients with a lower BMI and higher initial fitness may have found it easier to adhere to an exercise regimen and were therefore more likely to gain benefits from the programme. For this reason, changes in cardio‐pulmonary fitness parameters rather than absolute values were assessed, essentially making patients their own controls. Additionally, target exercise thresholds were calculated on an individual basis. It is also worth noting that patients who were more motivated at baseline may have been naturally more optimistic during treatment which may in turn have influenced HQRL scores. This could explain the higher levels of emotional function and HRQL scores in patients participating in the trial, particularly the intervention group. Patients who declined to participate in the study had statistically worse survival than either the intervention or control group and maybe the population in greatest need of prehabilitation. This highlights the importance of adapting programmes to suit individual patients to gain maximal adherence.

To date, 13 studies have examined the role of exercise in oesophagectomy patients with varying methodologies and outcomes [18, 19, 20, 21, 22, 23, 24, 25, 26, 27, 28, 29]. The studies have been small and heterogenous although one small RCT has suggested a range of benefits with prehabilitation [18]. None have demonstrated a survival advantage in treatment groups compared with controls. The majority have shown no significant difference between post‐operative length of stay or complications although three studies reported a reduction in the latter [21, 23, 25]. Hospital stay may not be the best surrogate marker of surgical outcome despite being commonly reported. It may be influenced by pre‐determined enhanced recovery pathways, subjective clinician decision‐making or a given patients' social circumstances rather than their true readiness for discharge. However, the importance of understanding the financial benefits of prehabilitation, such as length of stay, in justifying implementation of these services should not be underestimated.

The deleterious effect of neoadjuvant chemotherapy on cardio‐pulmonary fitness is well documented in patients undergoing treatment for oesophageal cancer [30, 31]. Levels of fitness deterioration observed in the present study were similar to other published studies [30, 31]. A recent meta‐analysis [32] confirmed that CPEX metrics can predict post‐operative mortality and some studies have linked poor CPEX performance with increased morbidity [31]. It is unlikely that any exercise programme will entirely negate or reverse the physical deterioration of patients undergoing chemotherapy, however, the blunting of the decline in the intervention group, as measured by VO_2peak_, is an encouraging result.

Sarcopenia and sarcopenic obesity improved in the intervention group of this study. The association between raised body mass index (BMI) and the incidence of upper gastrointestinal cancers is well established, and the association is particularly strong for oesophageal adenocarcinoma [33, 34]. Patients requiring oesophagectomy are thus likely to be obese and NAC is known to induce sarcopenia and worsen sarcopenic obesity [10]. There is a strong carcinogenic association of visceral adipose tissue enhancing tumorigenesis in epithelial tissue [35, 36]. Sarcopenia has been associated with increased rates of tumour growth, disease progression and tumour recurrence after surgery [37, 38, 39]. There is no data that suggests that exercise may play a role in improving oncological outcomes [40] and enhanced chemotherapy response [41]. Sarcopenia and obesity have also been shown to correlate with postoperative complications, morbidity and survival [6, 42, 43, 44]. It is therefore encouraging that NAC‐associated sarcopenia appears to be reduced in patients undergoing a structured exercise programme.

Poor health and mental well‐being are common in patients with oesophageal cancer [9], and neo‐adjuvant chemotherapy prior to oesophagectomy is known to exacerbate this [8]. Additionally, there appear to be associations between sarcopenia and anxiety, depression and poor HQRL [45, 46]. Studies have shown that increased levels of physical activity and exercise correlate with improved HRQL in patients with cancer [47]. Post‐surgical studies in breast cancer patients have shown that engaging in physical activity helps to manage the decline in HRQL after surgery [48]. The findings of the present study suggest that structured exercise during NAC may also help HRQL. Interestingly, in this study, patients in the intervention group experienced a significant decline in emotional function in the postoperative period, when the exercise intervention was stepped down to allow for post‐surgical recovery.

Lymphocytes provide a vital component of tumour‐related immunity [49, 50]. The correlation between sarcopenia and lymphopenia has been established in cross‐sectional studies [51]. Multiple studies have demonstrated a link between lymphopenia and poor outcomes in patients undergoing treatment for oesophageal cancer [49, 50]. One retrospective analysis of 307 patients found 5‐year cancer‐specific survival was 21.6% in patients with lymphopenia compared with 43.8% in those with normal lymphocytes (*p* = 0.004) [52]. Higher lymphocyte counts are associated with higher tumour response rates in patients undergoing neo‐adjuvant chemoradiotherapy for oesophageal cancer [53]. One study demonstrated a significantly lower complete pathological response rate and a higher recurrence rate in patients with treatment‐associated lymphopenia [54]. The link between exercise and an increase in peripheral lymphocytosis has been demonstrated [55]. In one study, 2 weeks of moderate to intense structured exercise resulted in an increased number of activated CD4^+^ helper T cells and CD8^+^ cytotoxic T cells as well as circulating concentrations of certain oncoregulatory cytokines (hepatocyte growth factor (HGF), IL‐4, MIP1β (VEGF and TNF) cells [55]. In the pre‐EMPT trial, patients in the intervention group experienced improved chemotherapy response (published separately) [56]. The observed significant changes in lymphocyte counts between intervention and control groups may offer a putative explanation for this observed difference.

In conclusion, the results of this non‐randomised trial suggest that a structured exercise programme during NAC may blunt the deterioration in cardio‐pulmonary fitness, reduce sarcopenia and improve lymphocyte counts of patients undergoing treatment for adenocarcinoma of the oesophagus. This study also suggests that structured exercise can improve aspects of patients' HRQL and response to chemotherapy. The benefits of exercise during neo‐adjuvant chemotherapy emphasise the importance of initiating prehabilitation at the beginning of the patient pathway.

## On Behalf of the Pre‐EMPT Study Group Including the Following Co‐Authors

J. Gossage, M. Kelly, C. Baker, J. Taylor, O. Rusu O. Evans, G. Tham (Department of Surgery, Guy's and St Thomas' Oesophago‐Gastric Centre, London, UK); T. Dixon, G. Hallward, C. Taylor (Department of Anaesthesia, Guy's and St Thomas' Hospital) N. Maisey, S. Ngan, A. Lumsden, K. Owczarczyk and A. Qureshi (Department of Oncology, Guy's and St Thomas Hospital, London, UK); N. Griffin and A. Jacques (Department of Radiology, Guy's and St Thomas' Hospital, London, UK); V. Goh (Cancer Imaging, School of Biomedical Engineering and Imaging Sciences, King's College Lon‐don, London, UK); M. Green, H. Deere, F. Chang, U. Mahadeva, B. Gill‐Barman, M. Ong, and S. George (Department of Pathology, Guy's and St Thomas' Hospital, London, UK); J. Dunn and S. Zeki (Department of Gastroenterology, Guy's and St Thomas' Hospital, London, UK) J. Waters, M. Cominos, T. Sevitt, M. Hill (Department of Oncology Maidstone and Tunbridge Wells and East Kent hospitals) A. Santa Ollala (Comprehensive Cancer Centre King's College London, United Kingdom), K. Beckmann and L. Gervais‐Andre (King's College London, UK), J. Pate (Centre for Health and Human Performance, London, UK).

## Conflicts of Interest

The authors declare no conflicts of interest.

## Synopsis of Paper

The Pre‐EMPT manuscript reports the results of a recent prospective trial assessing the impact of pre‐operative prehabilitation in patients with oesophageal malignancy treated on a peri‐operative pathway. This trial demonstrates that prehabilitation helps to mitigate cardiopulmonary deconditioning, reduces sarcopenia and offsets lymphopenia during chemotherapy in patients undergoing neoadjuvant chemotherapy and oesophagectomy for oesophageal adenocarcinoma.

## Supporting information

Supporting information.

Supporting information.

Supporting information.

## Data Availability

Data is available on request from the authors. (The data that support the findings of this study are available from the corresponding author upon reasonable request.)
