# Supplementary material for: Prehabilitation of Patients With Oesophageal Malignancy Undergoing Peri‐Operative Treatment (Pre‐EMPT): Outcomes From a Prospective Controlled Trial
Source: J Surg Oncol. 2025 Jan 29;131(8):1508–20. doi: 10.1002/jso.28079 (PMC12232078; doi:10.1002/jso.28079)
Supplement: Supplementary file 1 — Supporting information. [file JSO-131-1508-s001.docx]

**Supplementary Figure 1** CPEX outcomes at baseline, post neoadjuvant chemotherapy (NAC) and pre-surgery
