# Supplementary material for: Prehabilitation of Patients With Oesophageal Malignancy Undergoing Peri‐Operative Treatment (Pre‐EMPT): Outcomes From a Prospective Controlled Trial
Source: J Surg Oncol. 2025 Jan 29;131(8):1508–20. doi: 10.1002/jso.28079 (PMC12232078; doi:10.1002/jso.28079)
Supplement: Supplementary file 2 — Supporting information. [file JSO-131-1508-s002.docx]

**Supplementary Figure 2:** Changes in concentrations of CD3, 4, and 8 Lymphocytes at specified study timepoints (1. Baseline, 2. Post NAC, 3. Admission, 4. Day 1 post op, 5. Day 3, 6. Day 6, 7. 6 weeks)
