# Supplementary material for: Prehabilitation of Patients With Oesophageal Malignancy Undergoing Peri‐Operative Treatment (Pre‐EMPT): Outcomes From a Prospective Controlled Trial
Source: J Surg Oncol. 2025 Jan 29;131(8):1508–20. doi: 10.1002/jso.28079 (PMC12232078; doi:10.1002/jso.28079)

**Supplementary Table 1** EORTC questionnaire questions and their groupings

|  | Do you have any trouble doing strenuous activities, like carrying a heavy shopping bag or a suitcase? | Physical functioning |
| --- | --- | --- |
|  | Do you have any trouble taking a long walk? |  |
|  | Do you have any trouble taking a short walk outside of the house? |  |
|  | Do you need to stay in bed or a chair during the day? |  |
|  | Do you need help with eating, dressing, washing yourself or using the toilet? |  |
|  | Were you limited in doing either your work or other daily activities? | Role functioning |
|  | Were you limited in pursuing your hobbies or other leisure time activities? |  |
|  | Were you short of breath? | Dyspnoea |
|  | Have you had pain? | Pain |
|  | Did you need to rest? | Fatigue |
|  | Have you had trouble sleeping? | Insomnia |
|  | Have you felt weak? | Fatigue |
|  | Have you lacked appetite? | Appetite |
|  | Have you felt nauseated? | Nausea and Vomiting |
|  | Have you vomited? | Nausea and Vomiting |
|  | Have you been constipated? | Constipated |
|  | Have you had diarrhoea? | Diarrhoea |
|  | Were you tired? | Fatigue |
|  | Did pain interfere with your daily activities? | Pain |
|  | Have you had difficulty in concentrating on things, like reading a newspaper or watching television? | Cognitive Functioning |
|  | Did you feel tense? | Emotional Functioning |
|  | Did you worry? |  |
|  | Did you feel irritable? |  |
|  | Did you feel depressed? |  |
|  | Have you had difficulty remembering things? | Cognitive functioning |
|  | Has your physical condition or medical treatment interfered with your family life? | Social functioning |
|  | Has your physical condition or medical treatment interfered with your social activities? |  |
|  | Has your physical condition or medical treatment caused you financial difficulties? | Financial |
|  | How would you rate your overall health during the past week? | Quality of life |
|  | How would you rate your overall quality of life during the past week? |  |


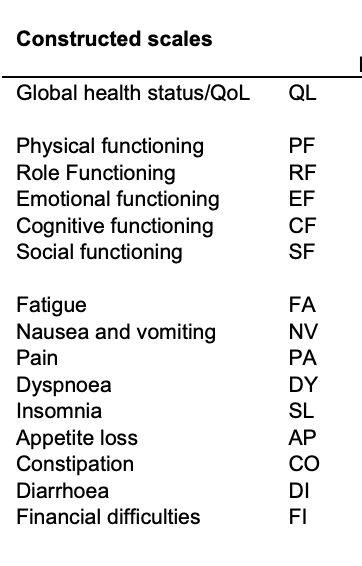

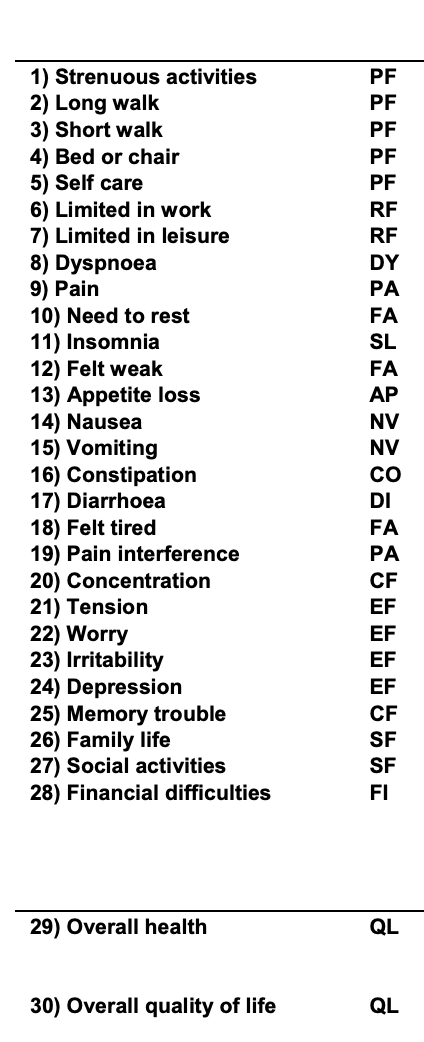

Supplement: Supplementary file 3 — Supporting information. [file JSO-131-1508-s003.docx]
